# Knowledge, Attitudes, and Motivations towards Blood Donation among King Abdulaziz Medical City Population

**DOI:** 10.1155/2014/539670

**Published:** 2014-11-06

**Authors:** Najd Alfouzan

**Affiliations:** Family Medicine Department, King Abdulaziz Medical City, National Guard Health Affairs, P.O. Box 22490, Riyadh 11426, Saudi Arabia

## Abstract

*Background*. Blood donation is remarkably safe medical procedure. However, attitudes, beliefs, and level of knowledge may affect it.* Objectives*. To measure the level of knowledge regarding blood donation, find out positive and negative attitudes, identify the obstacles, and suggest some motivational factors.* Methodology*. A cross-sectional study was conducted at King Abdulaziz Medical City (KAMC). Participants were selected by convenient nonrandom sampling technique. A self-created questionnaire was used for data collection.* Results*. The study included 349 individuals. About 45.8% of the participants claimed that they have a history of blood donation. Reported causes for not donating blood were blood donation not crossing their mind (52.4%), no time for donation (45%), and difficulty in accessing blood donation center (41.3%). Reported motivating factors for donating blood were one day off (81.4%), mobile blood donation caravans in public areas (79.1%), token gifts (31.5%), and finally paying money (18.9%).* Conclusion*. People in the age group 31–50 years, males, higher education and military were more likely to donate blood as well as People who showed higher knowledge level and positive attitude towards blood donation. More educational programs to increase the awareness in specific targeted populations and also to focus on some motivational factors are recommended.

## 1. Background

“More blood, more life,” this was the theme for World Blood Donor Day 2011 on the 14 of June to emphasize the critical need for more people all over the world to become lifesavers by donating blood regularly. Based on reports from 173 countries to WHO, around 93 million blood donors are donating annually [1].

Donated blood can be lifesaving for persons who have lost large amounts of blood because of serious accidents, new medical and surgical procedures, civil conflicts, and military wars as well as for patients who have become severely anemic because of serious hematological diseases or treatments such as cancer therapy. Therefore, availability of blood is an important concern to the society [2].

Over the last three decades, the source of blood has shifted dramatically from imported blood to locally recruited blood donors [3]. Currently, the sources of donated blood are involuntary donors (as a replacement for their relative's and friend's needs), voluntary unpaid donors, and paid donors.

Globally, higher rates of transfusion-transmitted infections have been documented among paid donors [4, 5]. Therefore, they are trying to reduce it as much as they can in many countries. In fact, the World Health Organization and the Council of Europe recommend that blood and blood components should only be collected from voluntary, unpaid repeat donors who can assist blood bank to manage blood supplies and schedule transfusion smoothly [6].

Based on the literature reviews, it can be stated that both developed and developing countries have problems with the unpaid blood donation system [7]. What encourages a person to donate blood for free? What are the obstacles facing a person? And how can the blood centers ask the donors to return again? Answers to these questions make it possible for blood collection agencies to determine which persons are expected to be new donors and enable making predictions of future donors [8].

Increase in the level of awareness and positive attitude towards blood donation is the highest priority of all blood transfusion centers. The initial step for achieving this goal is to perform comprehensive studies measuring the current situation of awareness, knowledge, beliefs, and both positive and negative attitudes of the population towards blood donation [9].

In this respect, studies continue to be published from different countries, developed [10–13] and developing [14–17], exploring the attitude and motivations of blood donors. The awareness and attitude were found to be different due to the difference in traditions, cultures, religion, and level of education. Therefore, in order for a blood donation system to be successful, it has to be in accordance with these elements of that society.

The most prominent reason why people give blood is altruism beside community needs and support, family assurances, and social pressure. On the other hand, fear, lack of knowledge, and inconvenience have been described to be the primary obstacles to donation.

## 2. Aim of the Study

The aim of the study is to explore the knowledge, attitudes, and motivations towards blood donation among Saudi population.

## 3. Specific Objectives

The objectives of the study are as follows:

1. to measure the level of knowledge regarding blood donation and its importance;
2. to find out positive and negative attitudes towards blood donation;
3. to identify the obstacles and difficulties facing the individuals;
4. to suggest some motivational factors that can improve the donation process in the future.

## 4. Materials and Methods

### 4.1. Study Area/Time

The study was carried out in King Abdulaziz Medical City (outpatients building) and two primary care health centers (Iskan and Kashmalaan), National Guard Health Affairs in Riyadh, Saudi Arabia, between 1 January 2013 and 1 March 2013.

### 4.2. Study Subjects

Any individual who has active file at KAMC and whose age is more than 16 years was included in our study. We excluded the medical staff.

### 4.3. Study Design

This is a cross-sectional, descriptive study.

### 4.4. Sample Size

After reviewing the population size from Registration Department at KAMC (~912,000), we calculated the sample.

Sample size is *n* = 350 with 95% confidence interval, alpha error of 0.05, accuracy of ±5%, and power of 80%.

### 4.5. Sampling Technique

Participants for this study were selected by convenient nonrandom sampling technique.

### 4.6. Data Collection Tool

A self-created questionnaire was used for data collection. It is a combination of three questionnaires used in previous studies in Saudi Arabia [2], China [4], and Iran [9]. Permission has been taken through emails from primary investigators. The questionnaire was validated by King Abdullah Research Center and two family medicine consultants expert in research methodology. It included questions covering 6 areas: demographic data, knowledge “9 questions,” attitude “4 questions,” practice “5 questions,” reasons for not donating, and motivation for donation. We assigned 2 physicians and 2 nurses to distribute the questionnaire to the participants. They explained unclear questions and helped illiterate individual to fill in the paper correctly. Questionnaire needed 10 minutes to be completed. Filled questionnaires were collected at the same time.

### 4.7. Study Variables

The questionnaire contains the following groups of variables:

- demographic data;
- knowledge about blood donation;
- attitude towards blood donation;
- practice of donations and its effects;
- motivations of blood donation.

*Pilot Study*. A pilot study has been conducted on 20 participants to evaluate data collection tool and methodology of study. Participants included in the pilot study were excluded from the main study.

### 4.8. Data Management and Analysis Plan

The Statistical Package for Social Sciences (SPSS version 20) was used for data entry and qualitative and quantitative statistical analysis. The descriptive statistics (frequency and percentage for categorical data during median and mean ranks for knowledge score) were computed. Since the total knowledge score is abnormally distributed (significant Kolmogorov-Smirnov “K-S test”), nonparametric statistical tests were applied. Mann-Whitney test was used for comparison of two groups and Kruskal-Wallis test was used for comparison of more than two groups. Chi-square was used to test for the difference between two categorical variables. Statistically significant differences were considered at *P* < 0.05.

Regarding knowledge score, right answers were given score “1” while wrong answers and do not know answers were given score “0.” Thus, the total knowledge score ranged between 0 and 9. while, for attitude score, the responses were scored in a way that the higher the score the more positive the attitude towards blood donation. Strongly agree was given a score “5,” agree “4,” neutral “3,” disagree “2,” and strongly disagree “1.” Thus the total attitude score ranged between 4 and 20.

### 4.9. Ethical Considerations

Family Medicine Research Committee and King Abdullah Research Center approved the research. There was a brief introduction in the first page of questionnaire assuring the confidentiality of individual's answers.

## 5. Results

### 5.1. Demographic Characteristics

The study included 349 Saudi individuals attending King Abdulaziz Medical City (KAMC) during the period of study. Their demographic characteristics are presented in Table 1.

### 5.2. Knowledge about Blood Donation

Almost two-thirds of participants (64.5%) knew their blood group type, while only 27.5% of them recognized the blood group type that can donate blood to any other needy individual. About one-third of them (33.2%) answered correctly that the minimum age for blood donation is 16 years, while 40.1% and 43.6% of them recognized correctly the minimum weight and the minimum interval between two times for blood donation, respectively. Most of the participants (80.5%) knew the location of blood bank in their community. Almost two-thirds of the participants (67%) thought that donating blood would not cause diseases or harm to the donor. Most of them (79.4%) acknowledged the blood bank screening of donated blood before transfusion and 59.9% knew that diabetic or hypertensive patients cannot donate blood.

### 5.3. Demographic Characteristics of the Participants and Their Knowledge about Blood Donation

#### 5.3.1. Age

As illustrated in Table 2, the highest level of knowledge about blood donation was reported among individuals in the age group of 20–30 years (mean rank = 186.7) compared to 119.9 reported among those aged less than 20 years. This difference was statistically significant, *P* = 0.004.

#### 5.3.2. Gender

Males had higher blood donation knowledge level rank compared to females in the study (mean rank was 191.9 versus 160.6), *P* = 0.003.

#### 5.3.3. Marital Status

Married individuals had higher blood donation knowledge level compared to singles (mean rank was 182.3 versus 158.9), *P* = 0.042.

#### 5.3.4. Level of Education

It is evident from Table 2 that the knowledge about blood donation level was increasing steadily with the increase in the educational level (the mean rank for knowledge score was highest among postgraduate individuals and lowest among illiterate individuals (229.6 versus 95.2), *P* < 0.001).

#### 5.3.5. Occupation

As demonstrated from Table 2, the highest level of knowledge about blood donation was reported among civil employees (mean rank = 212.1) while the lowest level was reported among students (mean rank = 127.1), *P* < 0.001.

### 5.4. Attitude towards Blood Donation

Most of the participants (78.6%) strongly agreed or agreed that blood donation is part of altruism, 71.3% strongly agreed or agreed that blood donation is a religious duty, 74.2% strongly agreed or agreed that blood donation is a national duty, and 81.9% strongly agreed or agreed that blood donation is a healthy habit.

### 5.5. Demographic Characteristics of the Participants and Their Attitude towards Blood Donation

#### 5.5.1. Age

As illustrated from Table 3, the highest level of attitude score towards blood donation was reported among individuals in the age group of 31–50 years (mean rank = 185.7) while the lowest level of attitude towards blood donation was observed among those aged below 20 years (mean rank = 141.0). However, this difference was not statistically significant, *P* > 0.05.

#### 5.5.2. Gender

Males had higher blood donation attitude score compared to females (mean rank was 197.5 versus 155.7), *P* < 0.001.

#### 5.5.3. Marital Status

Married individuals had higher blood donation attitude score compared to singles (mean rank was 184.6 versus 153.8), *P* = 0.007.

#### 5.5.4. Level of Education

As shown in Table 3, level of education was not significantly associated with attitude of the participant towards blood donation, *P* > 0.05.

#### 5.5.5. Occupation

As demonstrated from Table 3, the highest level of attitude score blood donation was reported among military individuals (mean rank = 204.6) while the lowest level was reported among students (mean rank = 130.4). This difference was statistically significant, *P* < 0.001.

### 5.6. Practice of Blood Donation

45.8% of the participants claimed that they have a history of blood donation.

### 5.7. Demographic Characteristics of the Participants and Their History of Practicing Blood Donation

#### 5.7.1. Age

More than half (56.8%) of individuals in the age group of 31–50 years compared to only 8.6% of those aged less than 20 years had a history of blood donation. This difference was statistically significant, *P* < 0.001.

#### 5.7.2. Gender

Males had significantly higher tendency towards donating blood compared to females (77.6% versus 18.6), *P* < 0.001.

#### 5.7.3. Marital Status

Married individuals had higher rate of blood donation compared to singles (53.3% versus 29.4), *P* < 0.001.

#### 5.7.4. Level of Education

The history of blood donation was steadily increasing with the increase in the educational level of the participants as 57.1% of individuals with postgraduate education compared to 20% of illiterates have donated blood previously, *P* = 0.007.

#### 5.7.5. Occupation

The majority of military personnel (89.7%) had donated blood compared to 18.6% of unemployed persons and 9.3% of students. This difference was statistically significant, *P* < 0.001.

### 5.8. Association between History of Blood Donation and Knowledge and Attitude

It is documented that blood donors had more knowledge regarding their blood group (*P* < 0.001), blood type that can be donated to any individual (*P* < 0.036), the minimum interval between two times of blood donations (*P* < 0.001), and location of blood bank in their communities (*P* < 0.001) than those with no history of blood donation. Regarding other areas of knowledge, there is no significant difference between both groups.

As shown in Table 4, individuals who had history of blood donation showed higher level of knowledge about it compared to those who did not have such history (mean ranks were 199.9 and 154, resp., *P* < 0.001).

More than half of blood donors (54.9%) compared to 45.1% of those who did not donate blood strongly agreed that blood donation is part of altruism, *P* = 0.005. Similarly, 58.3% of blood donors compared to 41.7% of those not donating blood strongly agreed that blood donation is a religious duty, *P* < 0.001. Almost two-thirds of blood donors (63.1%) compared to 36.9% of those who did not donate blood strongly agreed that blood donation is a national duty, *P* < 0.001, while 60.3% of blood donors compared to 39.7% of those who did not donate blood strongly agreed that blood donation is a healthy habit, *P* = 0.001.

Table 5 demonstrates that individuals who had history of blood donation showed higher attitude towards blood donation compared to those who did not have such history (mean ranks were 196 and 157.2, resp., *P* < 0.001).

### 5.9. Detailed History of Blood Donation

**Figure 1:**
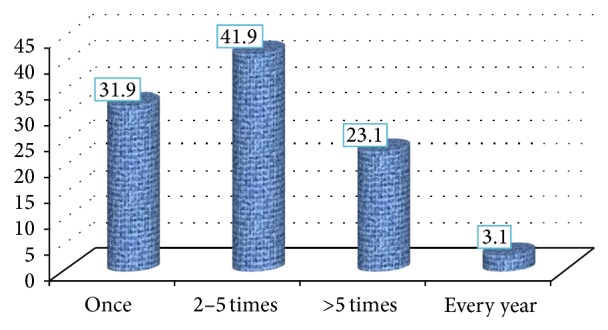
Frequency of blood donation among participants who had history of blood donation (*n* = 160).

Figure 1 shows the frequency of blood donation among participants who had history of blood donation.

More than half (55.6%) of individuals who donated blood reported that they did this voluntarily while 15.6% of them reported that they donated blood for their relatives or friends.

The majority of individuals who donated blood before (95.6%) reported that they will do it again and 96.3% of them described blood donation as a positive experience and they will encourage their friends to donate blood in the future.

Among those who had history of not donating blood before, almost one-third of them (33.9%) have mentioned that they had health problems preventing them from blood donation.

#### 5.9.1. Causes Mentioned by Participants for Not Donating Blood

More than half of them (52.4%) mentioned that blood donation did not cross their minds and 45% mentioned that they had no time for donation while 41.3% mentioned that they had difficulty in accessing blood donation center. More than one-third of them (37.6%) reported that they had fear of needle or seeing blood and 25.9% mentioned that blood donation procedure is a painful experience.

#### 5.9.2. Motivations Mentioned by Participants for Donating Blood

Most of them (81.4%) agreed that one day off is a motivational factor for donation and 79.1% of them agreed that mobile blood donation caravans in public areas (malls, plazas, and streets) are a good motivational factor for donating blood. Only 39.3% reported that media encourage people to donate blood very well, 31.5% agreed with token gifts, and 18.9% agreed with paying money as motivating factors for blood donation.

## 6. Discussion

Recruiting a sufficient number of safe blood donors in Saudi Arabia is an emerging challenge especially with the increase in demands as a result of an increase in population size and an increase in the number of medical facilities in Saudi Arabia. The present study has been conducted in Riyadh city in order to understand the various factors contributing to beliefs, attitudes, and level of knowledge associated with blood donation and transfusion that should help Saudi blood centers in building and maintaining an adequate and safe blood supply.

The current study results show a general lack of information regarding donation policies and practices among the surveyed individuals. As a group, donors had better understanding of the donation process. Although the experience of having donated blood likely explains why donors are more knowledgeable in this area, it is also possible that an increased availability of correct information on donation requirement to more eligible potential donors may help persuade some of them to donate.

Religion is deeply rooted in the Saudi society and there is little doubt that it is a major motivating factor for the local population to donate blood, as 71% of the donors in the current study believe that blood donation is a religious duty. A higher rate (91%) has been reported in another recent Saudi study [3]. This very high response rate may, in part, be based on the religious ruling [“fatwa”] from the most respected religious cleric, the late Sheikh Abdul Aziz bin Baz, who advised that it is the duty of a Muslim to donate blood to save the life of a needy patient; pamphlets carrying his “fatwa” are placed in most donor centers in Saudi Arabia.

In contrast, a Nigerian study [18] found that 20.3% of their study population would not donate blood and, curiously enough, will not accept blood transfusion due mainly to religious beliefs, a situation reminiscent of the behavior of Jehovah's witnesses [19, 20]. Thus the religious factor could have either a positive or negative motivating effect on blood donation. An active role of religion in improving the safety of donated blood which has recently been shown as blood donations collected at places of worship has greater chance of attracting donors free from transmitting HIV infection [21].

Other than the religious factor, the effectiveness of various incentives offered in return for blood donation has been highlighted in different studies and these include health-related incentives, such as blood credit, cholesterol, and PSA screening for donors older than 25 years, ticket to events, lottery, and/or raffle tickets for younger donors (<25 yrs.) [22], and health-related or economic incentives also confirmed in other studies [12, 23]. Our population is predominantly of middle-age groups (20–50 yrs.), and the positive motivational responses from both donors and nondonors were one day off and mobile blood donation caravans in public areas. In another Saudi study [3] the most motivating factor was token gifts; however, their population was younger in age. Other than incentives, effective measures have frequently been shown to encourage blood donation; such measures include inducing a “sense of give” among the public, when presented with hypothetical emotionally charged situations dramatizing the need for donor blood [21], “sense of solidarity or duty” and the possible personal or family benefits that donation might bring [24, 25], feeling of satisfaction, being more alert, and feeling generally better, after blood donation [10], as well as a sense of sharing and willing to accept the export blood to benefit other local communities in need [26]. A recent report from Nigeria [16] found that 41% of donors prefer certificates as incentives for donation. In the present study a high proportion of donors have a positive attitude toward blood donation as more than 95% of them would further donate if called upon and described their experience as a positive one.

Blood banks always follow screening guidelines and eligibility requirements to make sure that blood donation will not harm the donor [2]. In addition, new sterile disposable consumables are used for each donor to eliminate the risk of transmitting a blood-borne infection. Nonetheless, almost one-third of the participants thought that donating blood would cause diseases or harm to the donor.

Similarly, Sharma et al. found the same belief in most of their sample [27]. In Saudi Arabia, 11.5% of the participants in another study believed that blood donation is harmful to the donor [2]. In addition, Sastre et al. reported that French population has misconception regarding acquiring AIDS and hepatitis C infection as a result of donation [28]. Therefore, decreasing the perception that the blood donation is harmful can lead to an increase in the pool of blood donors.

It has been reported that age and gender are important identifiers of those less willing to donate [21, 29, 30]. Likewise, in this study donors were more likely to be males than females. In addition, more donors were between 30 and 50 years of age. Therefore, donor recruitment efforts should be directed towards age-gender groups with the lowest level of willingness to donate including females and those with the age range 16–30 years.

More than half (55.6%) of individuals who donated blood in the current study reported that they did this voluntarily while 15.6% of them reported that they donated blood for relatives or friends. In another Saudi study [3], the majority 84.5% of the participants preferred the donors to be direct donors either family members or friends to eliminate the risk of acquiring an infectious disease. In addition, 49% of the participants in that study stated that they would accept blood transfusion only from a relative. However, despite careful donors screening and blood testing, the incidence and prevalence of transfusion-transmitted infection are high in recipients receiving blood from direct donors and paid donors [31]. Therefore, Moltzan et al. showed that there is an increase in preoperative autologous blood donation in Canada due to increased concerns about allogenic blood safety [32]. In addition, Dhingra-Kumar et al. reported that autologous blood transfusion should be implemented in countries with high incidence of transfusion-transmitted infection to reduce the chance of transmitting blood-borne infectious agents and to increase blood banks supply [33]. Thus, the safest blood remains to be your own. When the blood transfusion is needed, the harm versus benefits is weighted carefully.

It is surprising to find that participants in the current study were not well informed about some areas of blood donation and blood transfusion in general as blood type that can be donated to any individual and minimum age and weight for blood donation as well as minimum interval between two times of blood donation. Proper knowledge of blood donation is an important factor for blood donation in the present study as more knowledgeable subjects tended to donate blood more than those of lower level of knowledge.

## 7. Conclusion

Knowledge regarding some aspects of blood donation is insufficient among the study population. However, their attitude towards it is generally satisfactory.

People in the age group of 31–50 years, males, and those of higher education and the military were more likely to donate blood while those under 20 years, females, those of lower education, and students were less likely to donate blood.

People who showed higher knowledge level of blood donation and those showing more positive attitude towards blood donation were more likely to donate blood.

Most reported motivating factors for blood donation were one day off and mobile blood donation caravans in public areas (malls, plazas, and streets).

## 8. Recommendations

1. Educational programs on blood donation and blood transfusion should be expanded through various media including the Internet to keep the topic of blood donation alive in the minds of the general public. These programs might focus more heavily on the benefits of blood donation and the idea that blood donation does not pose significant health risks.
2. The public should know that all measures besides screening tests are implemented by blood banks to ensure that blood donation is safe for donors and that transfusion of the donated blood is safe for recipients.
3. Increase in the level of awareness of women, young people, and students needs to be the topmost priority and barriers to donation by women, who comprise about 50% of society, should be evaluated by future studies.
4. More innovative factors for blood donation such as one day off and mobile donation caravans in public areas should be supported.

## Figures and Tables

**Table 1:** Demographic characteristics of the participants (*n* = 349).

Demographic characteristics	Percentage (%)
Age (years)	
<20	10.0
20−30	40.7
31−50	42.4
>50	6.9
Gender	
Males	46.1
Females	53.9
Marital status	
Single	31.2
Married	68.8
Level of education	
Illiterate	10.0
Below high school	14.9
High school	39.5
University	33.5
Postgraduate	2.0
Occupation	
Military	27.8
Civil	28.9
Students	15.5
Unemployed	27.8

**Table 2:** Association between demographic characteristics of the participants and their knowledge about blood donation.

Demographic characteristics	Blood donation knowledge		*P* value
	score (0–9)		
	Median	Mean rank	
Age (years)			
<20	4	119.9°	0.004^♦^
20–30	5	186.7^▪^	
31–50	5	179.0	
>50	4.5	161.8	
Gender			
Males	6	191.9	0.003^*^
Females	5	160.6	
Marital status			
Single	5	158.9	0.042^*^
Married	5	182.3	
Level of education			
Illiterate	4	95.2°	<0.001^♦^
Below high school	4	141.8	
High school	5	180.7	
University	6	203.7	
Postgraduate	7	229.6^▪^	
Occupation			
Military	6	195.6	<0.001^♦^
Civil	6	212.1^▪^	
Student	4	127.1°	
Unemployed	4	142.5	

^*^Mann-Whitney test.

^♦^Kruskal-Wallis test.

^▪^Highest mean rank.

°Lowest mean rank.

**Table 3:** Association between demographic characteristics of the participants and their attitude towards blood donation.

Demographic characteristics	Blood donation attitude		*P* value
	score (4–20)		
	Median	Mean rank	
Age (years)			
<20	15	141.0	0.117^♦^
20–30	16	173.2	
31–50	16	185.7	
>50	16	169.3	
Gender			
Males	16	197.5	<0.001^*^
Females	16	155.7	
Marital status			
Single	16	153.8	0.007^*^
Married	16	184.6	
Level of education			
Illiterate	16	149.2	0.320^♦^
Below high school	16	183.9	
High school	16	181.3	
University graduates	16	174.0	
Postgraduate	14	130.9	
Occupation			
Military	17	204.6	<0.001^♦^
Civil	16	179.6	
Student	15	130.4	
Unemployed	16	165.4	

^*^Mann-Whitney test.

^♦^Kruskal-Wallis test.

**Table 4:** Association between history of blood donation among participants and their total knowledge score about blood donation.

History of blood donation	Blood donation total knowledge		*P* value^*^
	score (0–9)		
	Median	Mean rank	
Yes	6	199.9	<0.001
No	5	154.0	

^*^Mann-Whitney test.

**Table 5:** Association between history of blood donation among participants and their total attitude score towards blood donation.

History of blood donation	Blood donation total attitude		*P* value^*^
	score (4–20)		
	Median	Mean rank	
Yes	16.5	196.0	<0.001
No	16	157.2	

^*^Mann-Whitney test.
